# Effect of Nitrogen Concentration on Growth and Fatty Acid Composition in the Freshwater Diatom *Stephanocyclus meneghinianus*

**DOI:** 10.3390/ijms27209030

**Published:** 2026-10-12

**Authors:** Anna Selezneva, Maria Gololobova, Zinaida Krivova, Elena Kezlya, Maxim Kulikovskiy, Alexander Starikov, Yevhen Maltsev

**Affiliations:** 1 K.A. Timiryazev Institute of Plant Physiology RAS, IPP RAS, 127276 Moscow, Russia; seleznevaanna03@gmail.com (A.S.); kosiapeya@mail.ru (Z.K.); melosira@mail.ru (E.K.); max-kulikovsky@yandex.ru (M.K.); starikovay1393@gmail.com (A.S.); 2 Faculty of Biology, M.V. Lomonosov Moscow State University, 119234 Moscow, Russia; gololobovama@mail.ru

**Keywords:** biotechnology, diatoms, fatty acids, microalgae, nitrogen, starvation

## Abstract

Microalgae can alter their growth rate and metabolic pathways in response to nutrient concentration in the medium, particularly nitrogen, one of their most important components. Novel freshwater diatom strains of *Stephanocyclus meneghinianus* (M418 and M456) were discovered in Moscow, Russia. Analysis was based on morphological characters, 18S rDNA and *rbc*L phylogeny, and the impact of changes in nitrogen concentration (from 0 to 0.6 g N/L) in WC medium on growth and total fatty acid composition. The results demonstrated that increasing nitrogen content in the nutrient medium stimulates culture growth. Strain M418 exhibited sustained exponential growth across all conditions, while strain M456 showed a clear transition to stationary phase, highlighting fundamental differences in their growth strategies. Principal component analysis underscored that strain M418 possesses a distinct and more strongly nitrogen-responsive fatty acid profile compared to strain M456. The main fatty acids for both strains were palmitic, palmitoleic, myristic, and eicosapentaenoic acids. The findings indicate that high nitrogen concentration is optimal for monounsaturated fatty acid accumulation, whereas nitrogen starvation stimulates that of polyunsaturated fatty acids.

## 1. Introduction

Diatoms are microscopic unicellular or colonial eukaryotic algae that inhabit freshwater and marine ecosystems as well as terrestrial habitats, and according to Guiry [1], there are approximately 18,000 species. Previously, diatom research focused mainly on fundamental questions of morphology, taxonomy, distribution, ecology, etc.; however, they now attract considerable attention because of their biotechnological potential. Diatoms synthesize various metabolites used in pharmaceuticals, biofuel production, and biofertilizers [2]. Scientists are therefore interested in studying diatoms since it makes it possible to find species and strains that are useful for biotechnology.

One of the diatom species of interest for biotechnology is *Stephanocyclus meneghinianus* (Kützing) Kulikovskiy, Genkal et Kociolek (=*Cyclotella meneghiniana* Kützing). *Stephanocyclus* Skabichevskij was resurrected by Kulikovskiy et al. [3] based on morphological differences in the valve margin structure and central area, the absence of a second wall layer characteristic of the genus *Cyclotella* (Kützing) Brébisson, and molecular data. In general, *S*. *meneghinianus* has been the subject of a comparatively considerable number of investigations. For example, the species can grow in both low and high salinity, indicating its euryhaline nature [4]. This species can synthesize fucoxanthin at a level of 7.76 mg/g dry weight [5]. Moreover, due to the high porosity of its frustules and the rounded shape of cells, *S*. *meneghinianus* is a promising candidate for use as a basis for drug delivery systems [6].

Nowadays, microalgae, including diatoms, are cultivated for a multitude of purposes worldwide. In 2020, the global microalgae market was valued at 3.4 billion USD and is projected to reach 4.6 billion USD by 2027. The growth is driven by increasing interest in sustainable development and awareness of the diversity of microalgal metabolites [7]. A key challenge for researchers in this biotechnology sector is selecting optimal cultivation conditions to enhance biomass accumulation. Numerous parameters influence both growth rates and biomass yield, as well as the accumulation of specific metabolites within the cells [8]. However, selecting a suitable strain and considering its individual characteristics when choosing cultivation conditions remain equally important [9].

The composition of the nutrient medium and the cultivation conditions directly affect microalgal cell growth and the buildup of intracellular compounds [10]. The nitrogen concentration in the medium is considered one of the most critical factors affecting microalgal growth, as this macronutrient is involved in the synthesis of proteins and nucleic acids [11]. Nitrogen deficiency in the nutrient medium slows culture growth rates [12] and alters the structure of the photosynthetic apparatus and cellular metabolism [13]. However, these changes are reversible, allowing restoration of photosynthetic function upon nitrogen repletion. Increasing nitrogen concentration in the nutrient medium enlarges chloroplasts and mitochondria within cells [13]. Conversely, at excessively high concentrations, nitrogen exerts a toxic effect on the cells, leading to deformation of chloroplast membranes, swelling and degradation of mitochondria, and cell plasmolysis [14].

Nitrogen starvation stimulates microalgae to synthesize lipids and complex carbohydrates, which serve as energy reserves necessary for surviving stressful conditions. It has been established that an increase in the synthesis of neutral lipids accompanies nitrogen deficiency. This is particularly relevant for the application of microalgae as a feedstock for biofuel production [15]. Alterations also extend to fatty acid composition; for instance, in the diatom *Phaeodactylum tricornutum* Bohlin, nitrogen starvation increases the proportion of polyunsaturated fatty acids (PUFAs) and decreases the amount of monounsaturated fatty acids (MUFAs) [12]. Sensitivity to nitrogen content is also characteristic of other algal groups. In strains of *Lobosphaera incisa* (Reisigl) Karsten et al. (Trebuxiophyceae), nitrogen limitation increased the intracellular arachidonic acid content [16]. Thus, on the one hand, nitrogen starvation causes microalgal cells to accumulate numerous metabolites that are beneficial for biotechnology; on the other hand, nitrogen deficiency restricts the amount of biomass produced. Consequently, the search for optimal cultivation conditions for microalgae for various biotechnological applications remains a relevant issue.

This study investigated the effect of different nitrogen concentrations in the nutrient medium on the growth and fatty acid composition of two strains of the centric diatom *Stephanocyclus meneghinianus*.

## 2. Results

### 2.1. Strain Description

#### 2.1.1. *Stephanocyclus meneghinianus* M418

**Figure 1:**
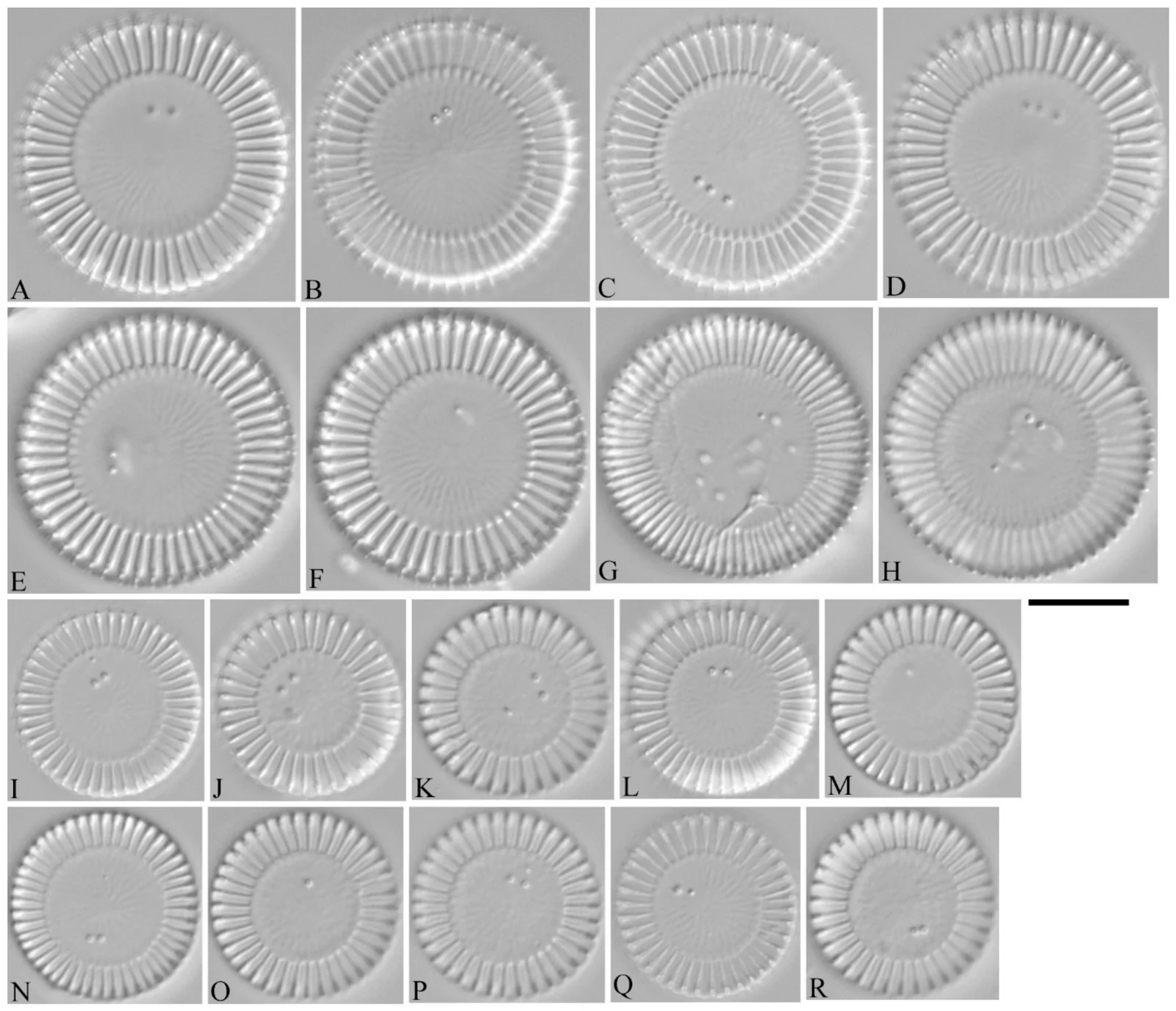
*Stephanocyclus meneghinianus*, LM, DIC, size diminution series. (**A**–**H**) Strain M418, (**I**–**R**) strain M456. Scale bar 10 μm (**A**–**R**).

Morphological description (Figure 1A–H). Cells are cylindrical in girdle view and circular in valve view. Cells are 28 to 30 µm in diameter, with one to two central fultoportulae. The valve margin striation is coarse, with 6–8 striae in 10 μm. In cultures, cells are solitary. Living cells are ochre in color due to their photosynthetic pigments.

Sequence data. GenBank accession PX678867 for the 18S rRNA gene partial sequence and GenBank accession PX685595 for the partial *rbc*L sequence.

#### 2.1.2. *Stephanocyclus meneghinianus* M456

Morphological description (Figure 1I–R). Cells are cylindrical in girdle view and circular in valve view. Cells are 19 to 21 µm in diameter, with one to three central fultoportulae. The valve margin striation is coarse, with 5–9 striae in 10 μm. In cultures, cells are solitary. Living cells are yellow–brown in color due to their photosynthetic pigments.

Sequence data. GenBank accession PX678869 for the 18S rRNA gene partial sequence and GenBank accession PX685597 for the partial *rbc*L sequence.

**Figure 2:**
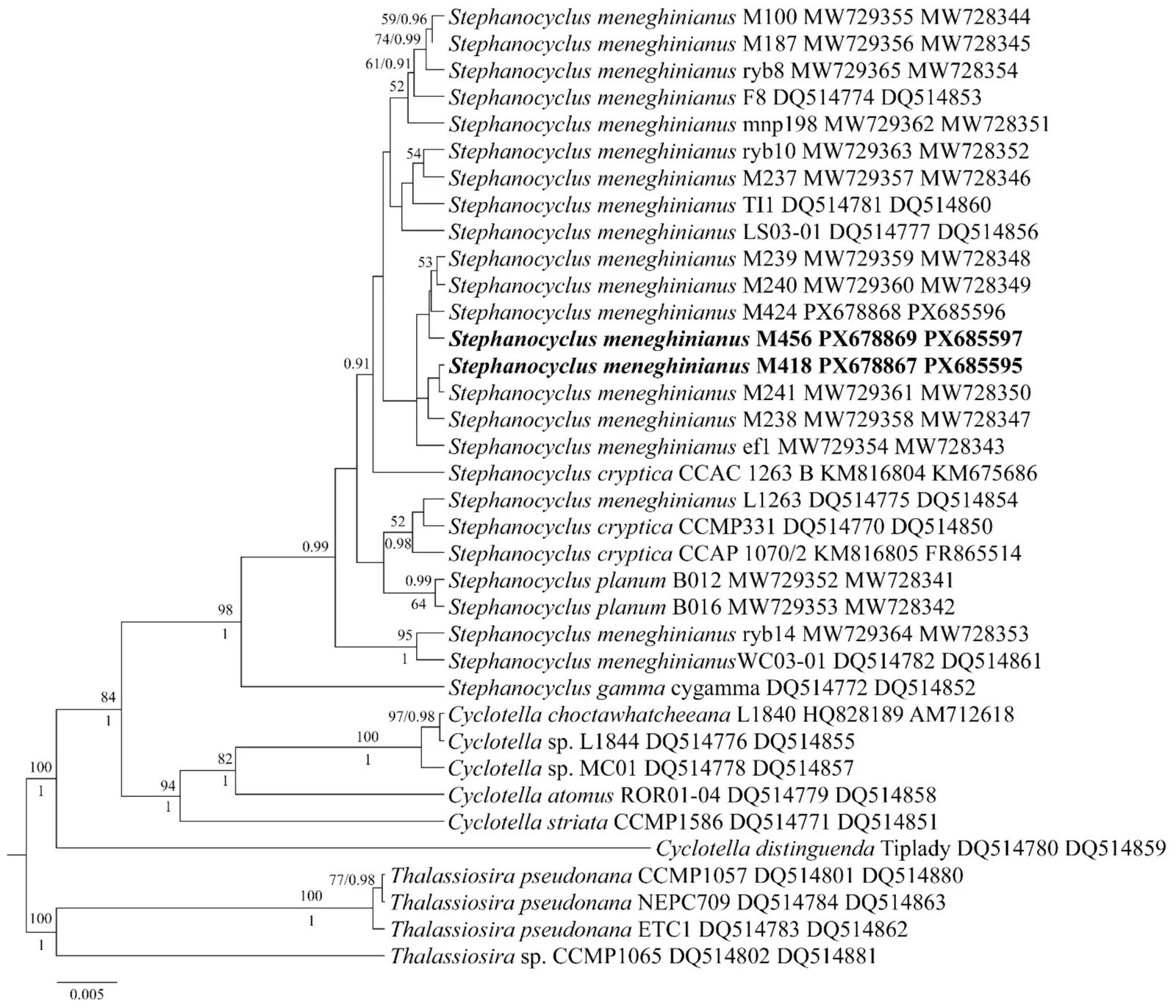
Phylogenetic position of the new *Stephanocyclus meneghinianus* strains (indicated in bold) based on Bayesian inference for the partial *rbc*L and 18S rRNA genes. The total length of the alignment is 1377 characters. Bootstrap supports (BSs) from ML (constructed by RAxML) and posterior probabilities (PPs) from BI (constructed by BEAST) are presented on the nodes in order. Only BS and PP above 50 and 0.9 are shown. Strain numbers (if available) and GenBank numbers are indicated for all sequences.

Molecular analysis. The 18S rDNA and *rbc*L phylogenetic tree was constructed with 30 nucleotide sequences of closely related centric diatoms derived from the National Center for Biotechnology Information (NCBI) and the sequences of the novel strains. All *Stephanocyclus* strains, including M418 and M456, formed one separate clade (Figure 2). Maximum Likelihood (ML) and Bayesian inference (BI) analyses gave congruent results for the phylogenetic placement, supported by high statistical values (Figure 2). M418 and M456 formed a common clade with other *S*. *meneghinianus* strains.

Both strains were classified as belonging to species *Stephanocyclus meneghinianus*, genus *Stephanocyclus*, family Stephanodiscaceae, order Stephanodiscales, class Mediophyceae, and phylum Heterokontophyta based on morphology and molecular data.

### 2.2. Growth Parameters

**Figure 3:**
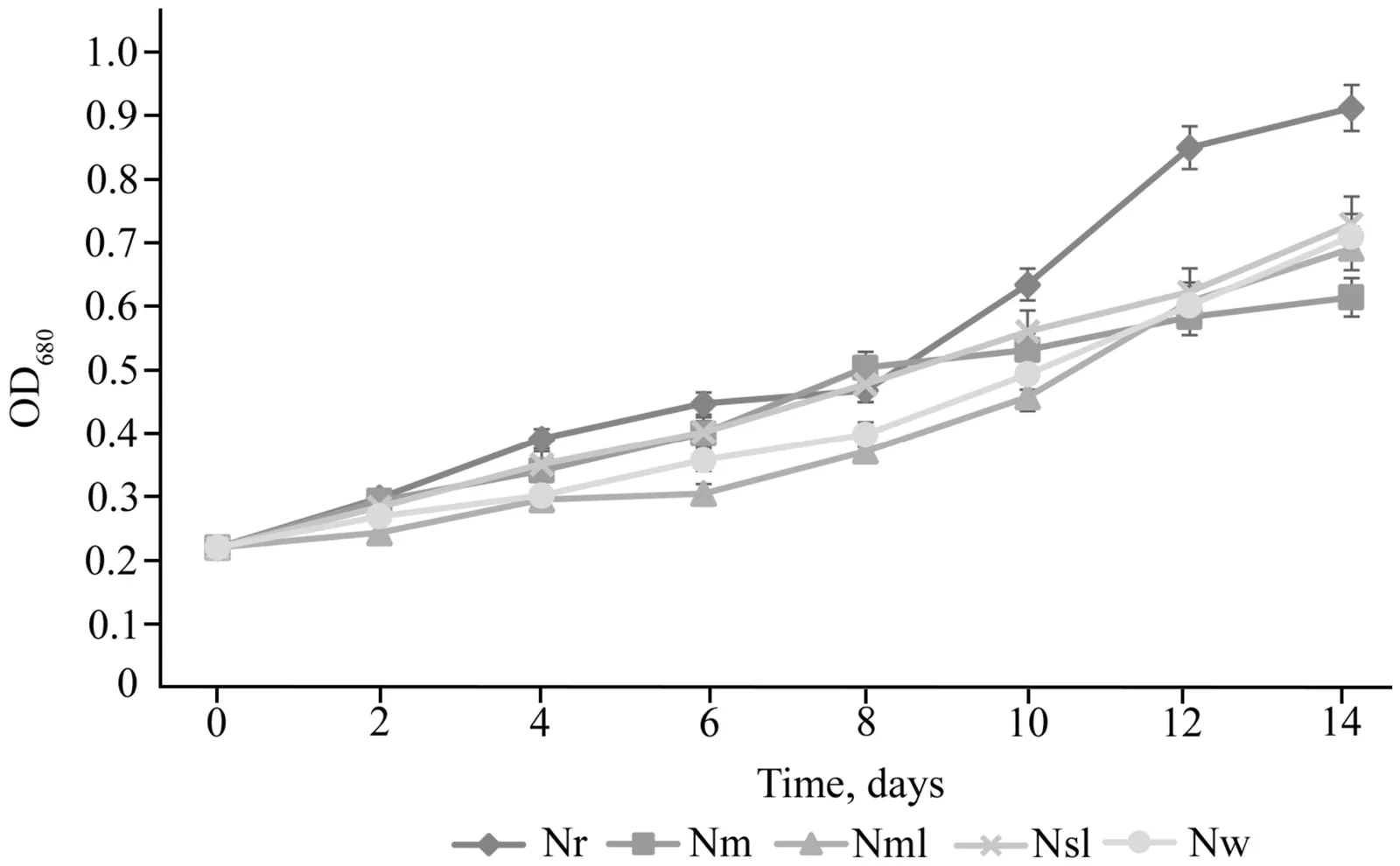
Growth curves of the strain *Stephanocyclus meneghinianus* M418 under different cultivation conditions based on the changes in the optical density (OD680). Values are mean ± SE (*n* = 3).

**Figure 4:**
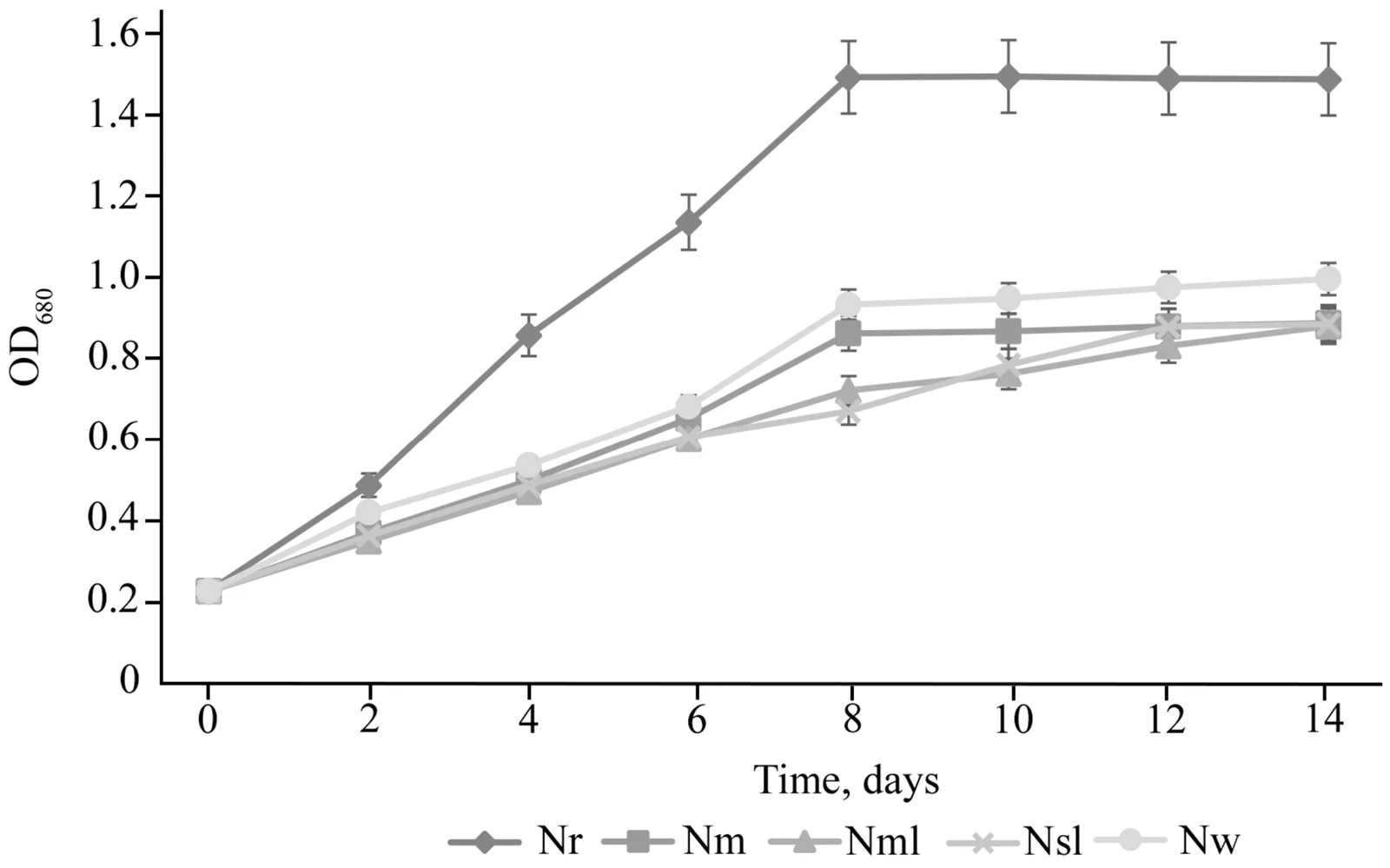
Growth curves of the strain *Stephanocyclus meneghinianus* M456 under different cultivation conditions based on the changes in the optical density (OD680). Values are mean ± SE (*n* = 3).

The influence of nitrogen stress on the growth and fatty acid composition of *S*. *meneghinianus* strains was studied using five modified variants of the WC medium, as presented in Table 1. Optical density measurements over the 14-day period revealed distinct growth patterns for the two strains (Table 2). Strain M418 remained in exponential phase throughout the experiments. Strain M456 exhibited exponential growth only until day 8, after which the cultures entered the stationary phase. The highest optical density at the end of the experiment (1.47 ± 0.01 for strain M456; 0.94 ± 0.01 for strain M418) was achieved in the nitrogen-replete medium (Nr). This value was significantly different (*p* < 0.01) from the values of the other cultures. Consequently, an increase in nitrogen concentration compared to its content in the standard WC medium stimulates the growth of *S*. *meneghinianus* (Figure 3 and Figure 4).

The growth dynamics of strain M456 in the Nm, Nsl, and Nml experimental variants were similar. The final optical density of these cultures ranged from 0.87 ± 0.01 to 0.88 ± 0.01. Cultures of strain M418 with nitrogen concentrations Nsl, Nw, and Nml also exhibited similar optical densities at the end of the experiment (ranging from 0.71 ± 0.01 to 0.75 ± 0.02). We assumed that the similar growth rates of the cultures on media with low and medium nitrogen content were due to the nitrogen concentration during the first cultivation stage on standard WC medium being close to that of the Nsl variant. On the 14th day of cultivation, M456 entered the stationary growth phase, as indicated by an OD680 of 0.87 ± 0.01. Conversely, on the same day, M418 remained in the exponential growth phase, with an OD680 of 0.75 ± 0.02.

Specific growth rate (μ) was calculated from measurements during the exponential growth phase. For the strain M456, μ was calculated from days 1–8, as it entered the stationary phase after day 8. This same period was selected for the strain M418 to allow for a direct comparison with strain M456. The specific growth rate for strain M418 ranged from 0.077 to 0.122 day^−1,^ with the maximum at moderate nitrogen content (Table 2). The strain M456 demonstrated a specific growth rate from 0.157 to 0.272 day^−1^, which was 2.2 times higher than M418. The highest mean was obtained in experimental variant Nr. At the end of the experiment, after 14 days of cultivation, strain M418 accumulated dry biomass ranging from 0.78 ± 0.04 to 1.12 ± 0.02 g/L, while strain M456 accumulated between 1.04 ± 0.05 and 1.69 ± 0.08 g/L. Thus, a nitrogen concentration of 0.6 g N/L proved to be optimal for biomass accumulation in the *S*. *meneghinianus* strains M418 and M456 (Table 2).

### 2.3. Fatty Acid Profiles

**Figure 5:**
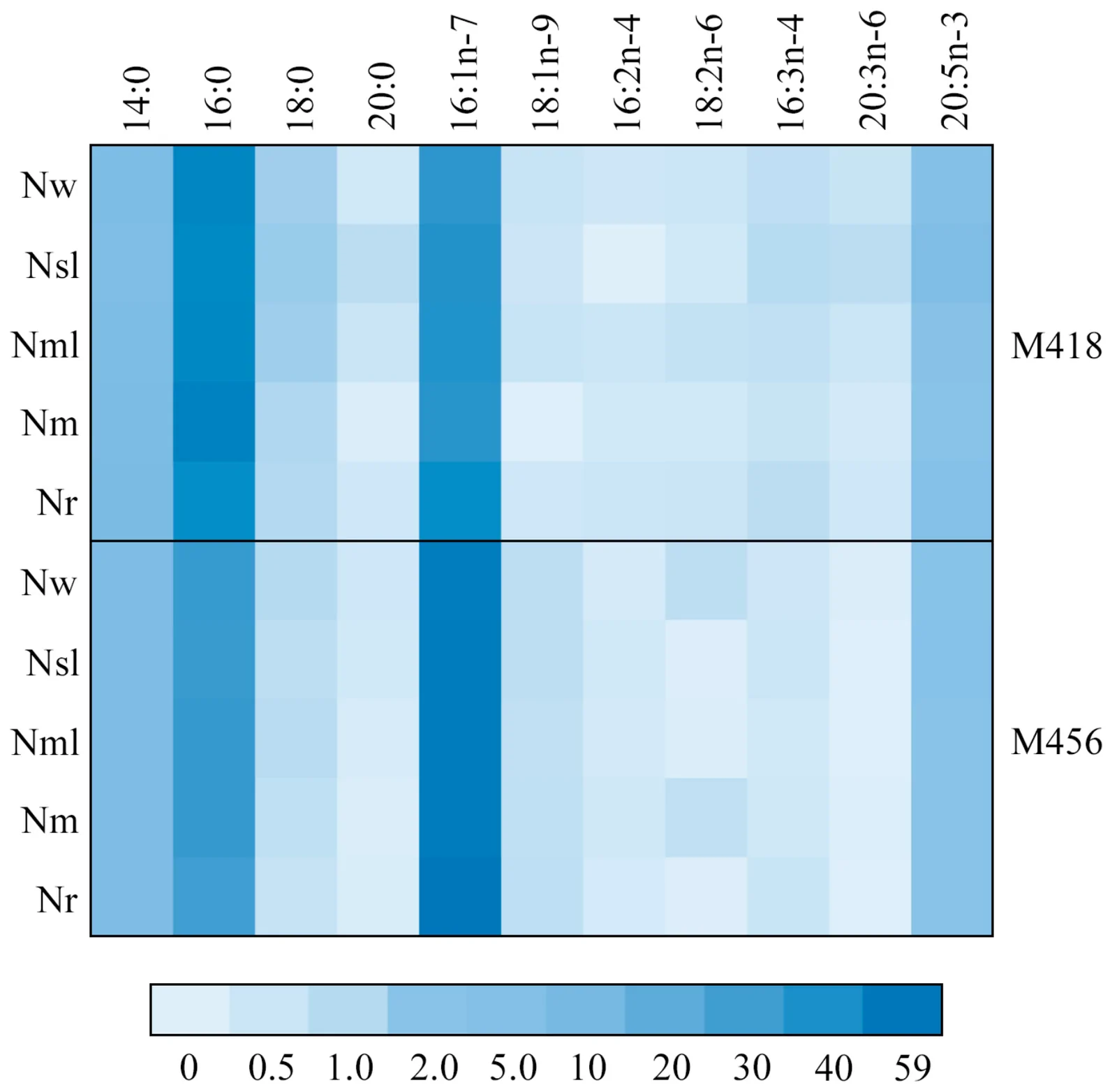
Fatty acid composition of *Stephanocyclus meneghinianus* (M418, M456) under different cultivation conditions. The color scale represents the fatty acid composition as a percentage of the total.

Fatty acid analysis of strains M418 and M456 revealed that the dominant fatty acids in both strains were palmitic (16:0) and palmitoleic (16:1n-7) acids. Myristic (14:0) and eicosapentaenoic (20:5n-3) acids were present in smaller quantities, while other fatty acids were found in trace amounts (Figure 5, Supplementary Table S1).

In cells of the M418 strain, saturated fatty acid (SFA) content ranged from 51.45 ± 0.37% (Nr) to 59.11 ± 0.53% (Nm). One-way analysis of variance (ANOVA) with Tukey’s post hoc test showed that nitrogen concentration in the nutrient medium significantly influenced palmitic acid content (Supplementary Table S1). Its level in the medium without nitrogen (47.82 ± 1.17%) differed from that in cultures with medium (50.49 ± 0.88%; *p* < 0.05) and replete (41.91 ± 0.66%; *p* < 0.01) nitrogen concentrations. Palmitic acid levels also differed significantly (*p* < 0.01) between the medium and replete nitrogen treatments. The MUFA content varied from 36.34 ± 0.38% (Nw) to 42.14 ± 0.46% (Nr). The amount of palmitoleic acid also varied significantly (*p* < 0.01) between the nitrogen-free media (35.73 ± 0.66%), strong limitation (38.41 ± 0.80%), and replete (41.72 ± 0.80%) nitrogen content in the media. The PUFA content ranged from 4.13 ± 0.09% (Nm) to 8.32 ± 0.12% (Nsl). The content of eicosapentaenoic acid in this strain differed among experimental treatments (*p* < 0.01 for most comparisons and *p* < 0.05 for Nw vs. Nr). The amount of omega-3 and omega-6 fatty acids in this strain was maximal under strong nitrogen limitation (6.13 ± 0.13% and 1.24 ± 0.06%, respectively).

In strain M456, the proportion of SFAs ranged from 36.83 ± 0.44% (Nr) to 41.49 ± 0.31% (Nml). Palmitic acid concentration in the nitrogen-replete culture was significantly lower (*p* < 0.01) than in the other treatments, which did not differ significantly from each other. The MUFA content ranged from 54.72 ± 0.55% (Nw) to 59.70 ± 0.52% (Nr). Palmitoleic acid also varied significantly (*p* < 0.01) between cultures grown in the nitrogen-replete medium (58.92 ± 0.90%) and the other experimental variants. The PUFA content varied from 2.97 ± 0.07% (Nml) to 4.26 ± 0.09% (Nw). The eicosapentaenoic acid (EPA) content did not differ between the nitrogen-free (2.57 ± 0.12%) and nitrogen-replete (2.46 ± 0.11%) treatments. A similar trend was observed between the moderate limitation (2.11 ± 0.09%) and medium nitrogen content (2.05 ± 0.09%) variants. The proportion of this fatty acid was maximal under strong nitrogen limitation (3.16 ± 0.15%) and differed significantly from the other treatments (*p* < 0.01). The maximum content of omega-3 and omega-6 fatty acids was observed under severe nitrogen starvation, consistent with the findings for strain M418.

Principal component analysis (PCA) was employed to analyze the effect of stepwise changes in nitrogen concentration in the nutrient medium on the fatty acid profiles of the strains M418 and M456. This method examined the original data to reveal new, uncorrelated variables (principal components, PCs). PCs allowed for the identification of broader underlying patterns. The analysis showed that, for this experiment, six principal components could be extracted. Among them, two key components collectively accounted for 69.15% of the total variance (Table 3).

**Figure 6:**
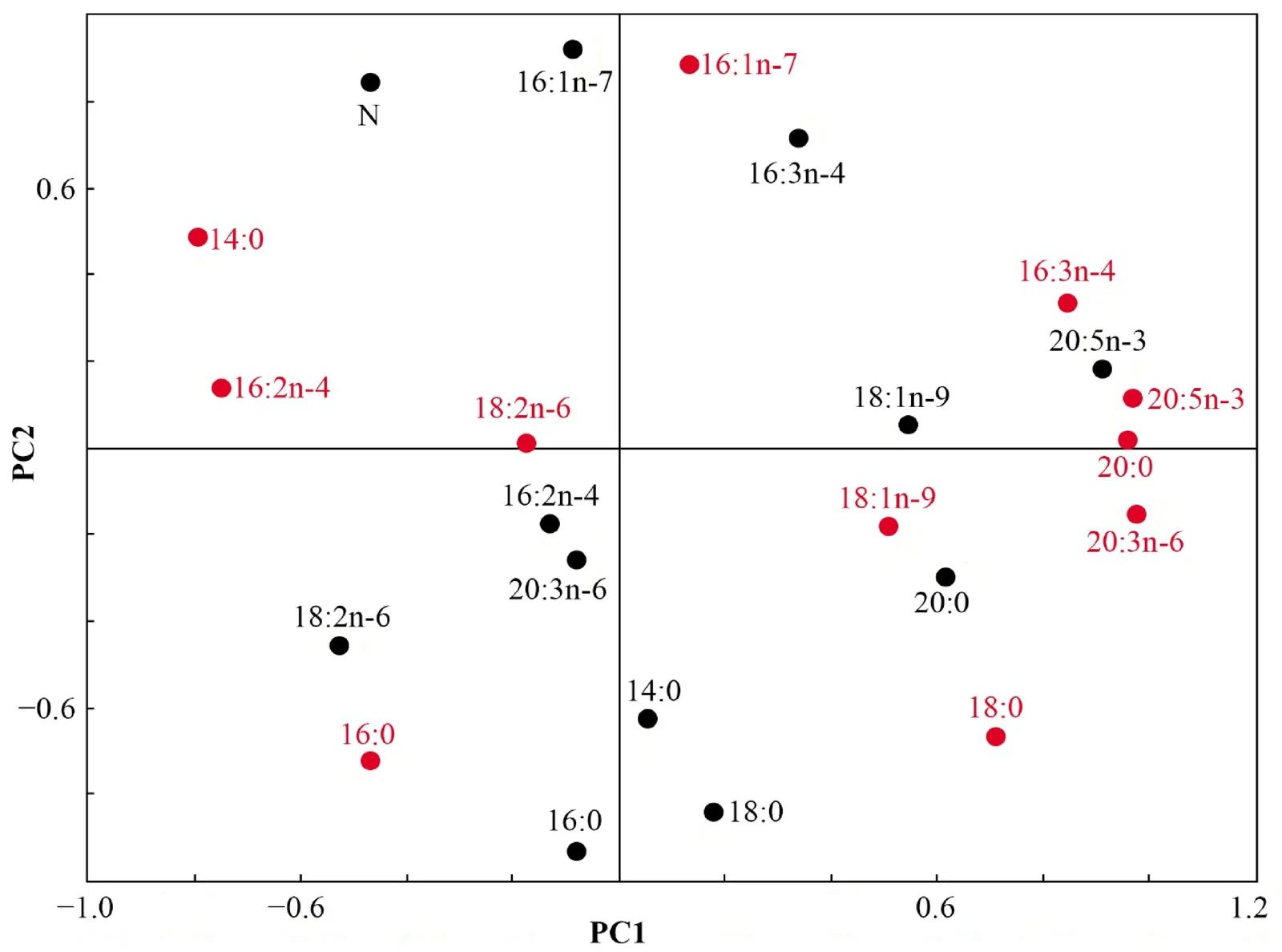
Projection of the fatty acid profiles of *Stephanocyclus meneghinianus* strains M418 and M456 under different cultivation conditions on the PC1–PC2 plane. Nitrogen (N), strain M418 (red markings), strain M456 (black markings).

PC1 accounted for 37.47% of the total variance. For strain M418, PC1 had strong positive loadings for myristic acid (14:0) and nitrogen concentration, and strong negative loadings for dihomo-γ-linolenic (20:3n-6), eicosapentaenoic (20:5n-3), stearic (18:0), arachidic (20:0), hexadecatrienoic (16:3n-4), and oleic (18:1n-9) acids. For strain M456, PC1 had strong negative loadings for arachidic (20:0), eicosapentaenoic (20:5n-3), and oleic (18:1n-9) acids, and moderate positive loadings for palmitoleic (16:1n-7) and hexadecadienoic (16:2n-4) acids. Thus, PC1 mainly contrasted myristic acid and nitrogen concentration with several PUFAs (particularly dihomo-γ-linolenic acid and EPA) in M418, and contrasted EPA, arachidic, and oleic acids with palmitoleic acid in M456 (Figure 6). Consequently, PC1 reflected the specific characteristics of the fatty acid composition of strain M418.

PC2 accounted for 28.85% of the total variance. PC2 had strong positive loadings for palmitic (16:0) acid in both strains, and for stearic (18:0) and linoleic (18:2n-6) acids in M456. It had strong negative loadings for palmitoleic (16:1n-7) acid in both strains, and moderate negative loadings for hexadecatrienoic acid (16:3n-4) and EPA. Therefore, PC2 mainly contrasted SFA (particularly palmitic and stearic acids) accumulation with palmitoleic acid and EPA (Figure 6).

## 3. Discussion

Analysis of morphological features of strains M418 and M456 showed a close relationship with species of the genus *Stephanocyclus*. The common features were cylindrical cells, presence of central fultoportulae, and the position of marginal fultoportulae.

The morphometric characteristics (in particular, valve diameter and number of striae) of the isolated strains coincide with those reported for this species in earlier descriptions [17,18,19]. At the same time, strain M456 was characterized by smaller cell sizes (up to 19–21 μm in diameter compared to 28–30 μm in strain M418) and a larger number of central fultoportulae (up to 3). Phylogenetic analysis by ML and BI methods using the 18S rRNA and *rbc*L genes showed that strains M418 and M456 were closely related to other *Stephanocyclus* strains, and first of all to the *S*. *meneghinianus* strains M239, M240, M241, and M424 collected in Moscow waterbodies (Figure 2). A sister position to *S*. *meneghinianus* strains was shown by the strain CCAP 1263 B *Stephanocyclus cryptica* (Reimann, Levin et Guillard) Kulikovskiy, Genkal et Kociolek (=*Cyclotella cryptica* Reimann, Lewin et Guillard). At the same time, *S*. *cryptica* is characterized by a smaller cell size (diameter 5–25 μm). In general, a similar phylogenetic tree topology was also obtained during a previous study addressing phylogeny questions for species from the Stephanodiscaceae [3,20,21].

Previous experience cultivating different *Cyclotella* and *Stephanocyclus* strains shows they can produce biomass ranging from 0.58 to 2.11 g/L. The lowest biomass yield was recorded for *S*. *cryptica* CCMP 331 cultured in heterotrophic conditions for 14 days on F/2 [22], whereas the highest yield was recorded for *Cyclotella cryptica* UTEX 1269 with 2.11 g/L after heterotrophic growth in 19 L carboys with an aeration rate of 0.25 v/v/min [23]. Consequently, the ability of M418 and M456 to produce biomass up to 1.69 g/L exceeds the average for most *Cyclotella* and *Stephanocyclus* strains after growth without aeration, including UTEX 1269 with 1.28–1.39 g/L in 250 mL Erlenmeyer flasks with agitation [23].

The physiological mechanisms by which nitrogen content influences diatom growth and fatty acid composition involve complex metabolic reallocations. Nitrogen status controls carbon partitioning through the glutamine synthetase/glutamate synthase (GS/GOGAT) pathway: under nitrogen deprivation, α-ketoglutarate (AKG) increases (up to 18-fold) while glutamate and glutamine pools decrease sharply. This metabolite ratio acts as a transcriptional signal, favoring carbon flux into lipids over proteins [24].

When cultivated in nutrient media with a stepwise gradient of nitrogen concentration, the *S*. *meneghinianus* strains M418 and M456 demonstrated the highest growth performance on nitrogen-rich variants of the WC medium (0.6 g N/L). Notably, strain M418 remained in exponential growth throughout the experiment, whereas strain M456 exhibited exponential growth only until day 8 before entering stationary phase. A similar result for the same diatom species was reported by Chinnappan et al. [5]. Their study also showed that increasing nitrogen content in the nutrient medium led to higher intracellular accumulation of fucoxanthin, β-carotene, chlorophylls, and carotenoids. A positive correlation between culture growth rates and nitrogen concentration in the nutrient medium is characteristic not only of the studied strains but also of many other species, such as *S*. *cryptica* [25] and *Phaeodactylum tricornutum* [26]. Conversely, excessively high nitrogen concentrations can inhibit algal growth. For instance, in *Chlamydomonas reinhardtii* P.A.Dangeard, an increase in sodium nitrate in the nutrient medium stimulated culture growth, but upon reaching a certain threshold, growth rates decreased [14]. Therefore, for the cultivation of microalgae for biotechnological purposes, it is necessary to identify a strain-specific range of optimal nitrogen concentrations.

Literature analysis indicates significant differences in the qualitative composition of fatty acids among diatoms. Taxonomic classification determines the fatty acid profile, which also varies with cultivation parameters, growth phase, and duration, with differences observable even between strains of a single species [27,28]. In general, diatoms are characterized by a high content of myristic (14:0), palmitic (16:0), palmitoleic (16:1n-7), and eicosapentaenoic (20:5n-3) acids [29]. At the class level, a key difference between Mediophyceae and Bacillariophyceae lies in their PUFA content. Specifically, roughanic (16:3n-3) and hexadecatrienoic (16:3n-4) acids are present in Mediophyceae but often absent in Bacillariophyceae [30].

Fatty acid analysis of the strains *S*. *meneghinianus* M418 and M456 revealed that palmitic (16:0), palmitoleic (16:1n-7), and myristic (14:0) acids constituted the majority (88.69–95.16%) of all fatty acids. The fatty acid profile is influenced primarily by strain specificity, as reflected in PC1 of the PCA. Differences in the fatty acid composition among strains of the same species have also been demonstrated for other diatom species, such as strains VP292 and VP289 of *Pinnularia microgibba* Kezlya, Y.Maltsev, Krivova et M.Kulikovskiy [31], and CBMCsvn749 and CBMCsvn749 of *Psammodictyon similis* Kapustin, Kezlya et Kulikovskiy [28]. A comparison of the profiles of the studied strains showed that M418 was richer in SFAs and PUFAs than M456, while M456 contained a higher amount of MUFAs. Compared to oils obtained from other strains of centric diatoms, oil produced by the strain *S*. *meneghinianus* M456 had a higher content of palmitoleic (16:1n-7) acid in the range 53.89–58.92%, than in the strain *Cyclotella* sp. with ~35–39% [22], *Cyclotella cryptica* UTEX 1269 with 31% [23] and *S*. *cryptica* CCMP 331 with 23.5–28.6% grown under autotrophic condition. On the other hand, CCMP 331 had a palmitoleic acid content similar to M456, 52.0–60.7%, after cultivation under heterotrophic conditions in the dark [32].

The fatty acid content was influenced by the nitrogen concentration in the nutrient medium, which showed a very strong correlation with PC2. Low nitrogen concentrations (Nw, Nsl) increased the proportion of SFAs. For example, in strain M456, the highest SFA content (41.02%) was observed in the nitrogen-free medium (Nw), largely due to an increase in palmitic acid (16:0). Conversely, low nitrogen conditions led to a decrease in MUFAs. The proportion of MUFAs (primarily palmitoleic acid, 16:1n-7) was lowest in the nitrogen-free or nitrogen-limited media and highest in the nitrogen-replete (Nr) medium. Rui et al. [33] reported similar results for *Conticribra weissflogii* (Grunow) Stachura-Suchoples et D.M.Williams. The low-nitrogen experimental condition played a key role in accumulating higher SFA content, including palmitic (16:0) acid up to 41.41% (Table 4). However, several studies [34,35] reported that nitrogen limitation in other diatom species not only increased SFA accumulation but also decreased the proportion of PUFAs (Table 4). Nitrogen starvation was accompanied by a decrease in PUFA content from 37.78 to 24.93% in *C*. *weissflogii* CCMP 1336 [34] and from 22.17 to 19.03% in *Chaetoceros muelleri* Lemmermann [35].

Given that the discussed strains were also collected from distinct habitats (freshwater M418, M456; marine CCMP 331, CCMP 1336, UTEX 1269; soil VP292, VP289), we propose that the variations in fatty acid content depend not only on the cultivation conditions, but also on the conditions of the original habitat. Similar conclusions were reached by Shaw et al. [36], who studied four strains of *Skeletonema costatum* (Greville) Cleve, isolated from different environments, and proposed that observed differences in the fatty acid content were driven by the strains’ origin. To investigate habitat-dependent variability, Peltomaa et al. [37] analyzed the fatty acid profiles and contents of 10 diatoms from marine, brackish, and freshwater environments. Results revealed that fatty acid profiles vary significantly by habitat, with freshwater strains exhibiting markedly higher omega-3 and omega-6 concentrations than brackish or marine counterparts.

The sum of SFAs and MUFAs in *S*. *meneghinianus* M418 and M456 biomass after different nutrient manipulations was in the range of 95.87–97.03%. Fatty acid structure affects key biodiesel properties, especially flash point, viscosity, oxidation stability, and low-temperature fluidity [38]. For biodiesel production, the most important fatty acids are those with short chains, which improve the cold-flow properties of the fuel, and saturated fatty acids, which ensure a high cetane number, thereby improving ignition quality [39]. Furthermore, MUFAs have been demonstrated to significantly enhance the cold-flow properties of biofuels [40]. Within the scope of our study, strain M418, due to its high SFA and MUFA content (over 90% in each experimental variant) with a predominance of palmitic acid (16:0), proved the most suitable candidate for biodiesel production. In comparison with other studies, the average concentration of palmitic acid in this strain is higher than that reported for *Phaeodactylum tricornutum* and *Conticribra weissflogii* [27] or *Thalassiosira pseudonana* Hasle et Heimdal [41]. We suggest that the strain *S*. *meneghinianus* M418 can be used as a source of fatty acids for biodiesel production.

Omega-3 and omega-6 fatty acids are essential components of the human diet and support the health of the cardiovascular and nervous systems [42]. Omega-6 fatty acids in the studied strains were relatively low (0.19–1.24%), while omega-3 fatty acids were higher (2.05–6.13%). Omega-3 fatty acids in strain M418 were higher than in strain M456 and reached their maximum (6.13%) under severe nitrogen limitation. Consequently, strain M418, especially when cultivated under controlled nitrogen limitation, is a promising candidate not only as a biodiesel feedstock (due to its high SFA) but also for co-producing valuable nutraceuticals, improving the overall economic viability of the cultivation process.

## 4. Materials and Methods

### 4.1. Microalgae Material

Two strains of centric diatoms (M418 and M456) obtained from the Culture and Barcode Collection of Microalgae and Cyanobacteria ‘Algabank’ (WDCM1318) of the K.A. Timiryazev Institute of Plant Physiology, Russian Academy of Sciences were studied. The geographic location of sampling sites with measured ecological parameters is presented in Table 5.

Preparation of microscope slides and microscope observations were carried out according to Kulikovskiy et al. [3]. Strains for light microscopy (LM) were processed by means of a standard procedure involving treatment with 10% HCl and concentrated H2O2. The material was washed with distilled water. Permanent diatom preparations were mounted in Naphrax. LM observations of the monocultures were performed using a Zeiss Scope A1 light microscope (Carl Zeiss Microscopy GmbH, Göttingen, Germany) equipped with an oil immersion objective (×100/n.a 1.4, DIC).

### 4.2. Molecular Study

Total DNA from the studied strains M418 and M456 was extracted using Chelex 100 Chelating Resin of molecular-biology grade (Bio-Rad Laboratories, Hercules, CA, USA). Nuclear gene 18S rDNA and plastid *rbc*L gene were amplified. For the highly variable V4 region of 18S rDNA (399 and 404 bp), D512f and D978rev primers were used [43]. The plastid *rbc*L (966 and 972 bp) was amplified using rbcL404+ [44] and dp7- [45] primers. Amplifications were carried out using PCR mastermixes (ScreenMix, Evrogen, Moscow, Russia). The amplification conditions for the 18S rDNA were as follows: initial denaturation for 5 min at 95 °C followed by 35 cycles of 30 s denaturation at 94 °C, 30 s annealing at 52 °C, and 50 s extension at 72 °C, with a final extension for 7 min at 72 °C. The amplification conditions for the *rbc*L gene were as follows: initial denaturation for 4 min at 94 °C followed by 44 cycles of 50 s denaturation at 94 °C, 50 s annealing at 53 °C, and 80 s extension at 72 °C, with a final extension for 10 min at 72 °C.

PCR products were visualized by horizontal electrophoresis in a 1.0% agarose gel stained with SYBR™ Safe (Life Technologies, Carlsbad, CA, USA). The products were purified with a mixture of FastAP, 10× FastAP Buffer, Exonuclease I (Thermo Fisher Scientific, Waltham, MA, USA), and water. The sequencing was performed using a Genetic Analyzer 3500 instrument (Applied Biosystems, Waltham, MA, USA).

Editing and assembling of the consensus sequences were carried out by processing the direct and reverse chromatograms in Ridom TraceEdit ver. 1.1.0 and Mega ver. 7 software [46]. The reads were supplemented with GenBank-extracted sequences of 34 centric diatom species (taxa names and Accession Numbers are given in Figure 2). Four *Thalassiosira* Cleve strains were used as an out-group. The 18S rDNA and *rbc*L sequences were aligned separately using the G-INS-i algorithm in the MAFFT ver. 7 software (RIMD, Osaka, Japan) [47]. The resulting data set comprised 405 and 972 nucleotide sites for nuclear 18S rDNA and plastid *rbc*L regions, respectively. The aligned 18S rRNA gene sequences were combined with the *rbc*L gene sequences into a single matrix for the concatenated 18S rDNA and *rbc*L tree.

The data set was analyzed using the BI method implemented in BEAST ver. 1.10.1 software [48] to construct a phylogeny. For the alignment, the most appropriate substitution model, shape parameter α, and a proportion of invariable sites (pinvar) were estimated using the Bayesian information criterion (BIC), as implemented in jModelTest ver. 2.1.10 [49]. This BIC-based model selection procedure selected the GTR + I + G model, pinvar = 0.4610, and α = 0.4130. A Yule process tree prior was used as a speciation model. The analysis ran for 5 million generations with chain sampling every 1000 generations. The parameters-estimated convergence, effective sample size (ESS), and burn-in period were checked using Tracer ver. 1.7.1 software [48]. The initial 25% of the trees were removed, and the rest were retained to reconstruct a final phylogeny. The phylogenetic tree and posterior probabilities of branches were obtained based on the remaining trees, which had stable estimates of the parameter models of nucleotide substitutions and likelihood. ML analysis was performed using RAxML ver. 7.0.4 software [50]. Nonparametric bootstrap analysis with 1000 replicas was conducted. FigTree ver. 1.4.4 software and Adobe Photoshop CC ver. 19.0 were used for viewing and editing the tree.

### 4.3. Design of the Experiment

A two-stage cultivation was performed in the study. The first stage aimed to acclimate the cultures to larger cultivation volumes. For this purpose, the strains were cultivated for 4 weeks in 40 mm Petri dishes containing 5 mL of standard WC medium [51]. Subsequently, the cultures were transferred to 60 mm Petri dishes containing 10 mL of medium and maintained for 3 weeks. Following this, the strains were cultivated for 4 weeks in 250 mL Erlenmeyer flasks containing 90 mL of nutrient medium until the cultures reached the stationary growth phase.

To investigate the effect of nitrogen stress on the growth of strains M418 and M456, five modifications of the WC medium with different nitrogen concentrations were prepared: 0 g N/L, 0.05 g N/L, 0.15 g N/L, 0.3 g N/L, and 0.6 g N/L (Table 1), which were selected based on the ranges established from an analysis of nitrogen concentrations in various nutrient media [11]. Nitrogen was supplied as NaNO_3_; concentrations are expressed as elemental nitrogen (g N/L). Thus 0.6 g N/L corresponded to 3.64 g/L NaNO_3_. The original WC medium contained 0.0851 g/L NaNO_3_.

During the second cultivation stage, strains M418 and M456 were grown for 2 weeks in 250 mL flasks with 90 mL of the modified WC nutrient medium (with varying nitrogen levels) and 10 mL of inoculum. Cultivation was carried out on ELMI Sky Line Shaker S-3L orbital shakers (ELMI Ltd., Riga, Latvia) with constant agitation at 75 rpm. The light intensity was set at 100 µmol photons/(m^2^ s) with a 16:8 light:dark photoperiod. Light intensity and color temperature were measured using a Sekonic C-800 spectrometer (Sekonic Corporation, Tokyo, Japan). Cultivation was maintained at 24 °C. The growth dynamics of the M418 and M456 strains were monitored by measuring the optical density of the culture suspensions at λ = 680 nm (OD680) using an IMPLEN Nanophotometer P300 (Implen GmbH, München, Germany). For optical density measurements, 1 mL of the culture suspension was sampled in triplicate. Throughout the cultivation period, cultures were examined using an Olympus CKX53 light microscope (Olympus Corporation, Tokyo, Japan) to confirm the absence of contamination. The dry weight (g) of the biomass per litter was estimated using a gravimetric method [52].

The specific growth rate (μ) was calculated using the following formula [53]:

μ (day^−1^) = (ln OD_1_ − ln OD_0_)/(t_1_ − t_0_),

where OD_1_ and OD_0_ are the optical density on day 1 (t_1_) and day 0 (t_0_).

### 4.4. Total Fatty Acid Composition Analysis

For fatty acid methyl ester (FAME) profiling, the biomass of the cultured strains was prepared according to Krivova et al. [54] and Gabrielyan et al. [55]. Culture suspensions were transferred to 50 mL tubes, washed free of the nutrient medium with distilled water, and pelleted by centrifugation for 3 min at 3600× *g*, after the supernatant was removed. The steps to remove the nutrient medium from the biomass were repeated three times. The resulting biomass was transferred into 2.0 mL vials, 0.4 mL of ethanol was added, and the mixture was stored.

For the saponification reaction, 0.1 mL of a 5 M aqueous KOH solution was added to the cells in alcohol, and the mixture was incubated for 60 min at 65 °C. The samples were washed twice with an equal volume of *n*-hexane (Khimmed, Moscow, Russia) to remove unsaponified components. The resulting solution contained soluble potassium fatty acid salts. A few drops of 20% sulfuric acid (Khimmed, Moscow, Russia) were added to shift the pH to slightly acidic, converting the fatty acids from salts to free fatty acids. Extract the free fatty acids with an equal volume of *n*-hexane. The resulting hexane solution was transferred to a clean vial and evaporated to dryness. Then, 0.15 mL of a 1% sulfuric acid solution in methanol was added to the dry residue. The sample was incubated at 50 °C for 30 min. Subsequently, 0.05 mL of distilled water and 0.5 mL of hexane were added to the sample. The mixture was vortexed and centrifuged. The FAMEs contained in the hexane phase were transferred to a clean vial and used for GC-MS analysis.

The resulting mixtures were analyzed using an Agilent 7890A gas chromatograph (Agilent Technologies, Santa Clara, CA, USA) equipped with an Agilent 5975C mass spectrometric detector. A DB-23 capillary column (Ser. no. US8897617H, Agilent Technologies, Santa Clara, CA, USA) (60 m in length and 0.25 mm in diameter) was used for the analysis. Heptadecanoic acid (Sigma-Aldrich, St. Louis, MO, USA) was used as an internal standard for fatty acid determination. Analysis conditions: helium carrier gas with a flow rate of 1 mL/min; injection volume of 1 µL; split flow ratio of 1:5; and an injector temperature of 260 °C. The temperature gradient program was as follows: from 130 °C to 170 °C at a rate of 6.5 °C/min; from 170 °C to 215 °C at a rate of 2.5 °C/min, hold at 215 °C for 25 min; from 215 °C to 240 °C at a rate of 40 °C/min, and a final hold at 240 °C for 50 min. The mass spectrometric detector operating temperature was 240 °C, and the ionization energy was 70 eV.

### 4.5. Statistical Analysis

The experiment and all measurements were performed in triplicate. Data were presented as mean ± standard error. Two-way analysis of variance (ANOVA) was used to assess the effects of strain, nitrogen concentration, and their interaction on growth and fatty acid variables. When the interaction was significant, one-way ANOVA followed by Tukey’s post hoc test was performed separately for each strain. PCA was performed on standardized variables: fatty acid percentages and nitrogen concentration. Principal components with eigenvalues > 1 were retained. Loadings were interpreted as correlations between variables and PCs; absolute loadings > 0.7 were considered strong. All analyses were performed with STATISTICA software ver. 7.0. The growth curves were constructed in Microsoft Excel ver. 1903 software (Microsoft Office, Redmond, WA, USA).

## 5. Conclusions

Thus, the regulation of nitrogen concentration in the nutrient medium is a flexible tool for manipulating the fatty acid profile of microalgae. Nitrogen limitation reduced the growth rates of *Stephanocyclus meneghinianus* strains M418 and M456. After 14 days of cultivation, M418 accumulated up to 1.12 g/L of dry biomass, and M456 reached 1.69 g/L. However, these conditions were favorable for the accumulation of saturated fatty acids and a reduction in monounsaturated fatty acid content within the cells. Furthermore, under severe limitations, a maximum cellular content of omega-3 fatty acids was observed. The biotechnological potential of strains depends on their strain-specific fatty acid composition: strain M418 is a promising candidate for biodiesel production. In contrast, strain M456 is a source of monounsaturated fatty acids. Our results show that combining strain selection with stress conditions enables production of microalgal biomass tailored to specific biotechnological purposes.

## Figures and Tables

**Table 1:** Concentrations of nitrogen in the WC medium for different experimental variants.

Designation	Experimental Variant	Range of Nitrogen, g N/L	Used Concentration, g N/L
Nr	replete nitrogen	>0.4	0.6
Nm	moderate nitrogen content	0.2–0.4	0.3
Nml	moderate limitation by nitrogen	0.1–0.19	0.15
Nsl	strong limitation by nitrogen	<0.1	0.05
Nw	without nitrogen	0	0
WC	original		0.0543

**Table 2:** Changes in optical density (OD680) of cultures during the experiment, specific growth rate, and final biomass yield. Values are mean ± standard error (SE) (*n* = 3).

Strain	Experimental Variant	1st Day	8th Day		14th Day	
		OD680	OD680	μ, day^−1^	OD680	Biomass, g
M418	Nr	0.22 ± 0.01	0.48 ± 0.01 ^AaD^	0.111 ± 0.004 ^A^	0.94 ± 0.01 ^A^	1.12 ± 0.02 ^A^
	Nm		0.52 ± 0.01 ^A^	0.122 ± 0.006 ^B^	0.63 ± 0.02 ^aBD^	0.78 ± 0.04 ^B^
	Nml		0.38 ± 0.01 ^B^	0.077 ± 0.003 ^C^	0.71 ± 0.01 ^bCD^	0.87 ± 0.04 ^C^
	Nsl		0.49 ± 0.01 ^A^	0.114 ± 0.005 ^A^	0.75 ± 0.02 ^C^	0.91 ± 0.04 ^C^
	Nw		0.41 ± 0.01 ^bBCD^	0.087 ± 0.004 ^C^	0.73 ± 0.01 ^C^	0.89 ± 0.03 ^C^
M456	Nr	0.22 ± 0.01	1.48 ± 0.02 ^A^	0.272 ± 0.013 ^A^	1.47 ± 0.01 ^A^	1.69 ± 0.08 ^A^
	Nm		0.85 ± 0.02 ^aBF^	0.193 ± 0.009 ^B^	0.88 ± 0.01 ^B^	1.07 ± 0.04 ^Ba^
	Nml		0.71 ± 0.01 ^bCF^	0.167 ± 0.08 ^Ca^	0.87 ± 0.01 ^BC^	1.06 ± 0.04 ^Ba^
	Nsl		0.66 ± 0.02 ^CD^	0.157 ± 0.007 ^Cb^	0.87 ± 0.01 ^BD^	1.04 ± 0.05 ^B^
	Nw		0.92 ± 0.04 ^BE^	0.204 ± 0.011 ^D^	0.98 ± 0.01 ^E^	1.16 ± 0.06 ^Cb^

Different superscript letters show significant differences: A–F Means within the same age of the strain are significantly different at *p* ≤ 0.01. a–b Means within the same age of the strain are significantly different with *p* ≤ 0.05.

**Table 3:** Matrix of factor loads of the studied variables for the strains M418 and M456.

	M418		M456	
Variable	PC1	PC2	PC1	PC2
14:0	0.7898	−0.1843	−0.2746	0.4851
16:0	0.2046	0.8934	−0.1731	0.8715
18:0	−0.8905	0.3434	−0.5709	0.7184
20:0	−0.7942	−0.3985	−0.8786	0.1159
16:1n-7	0.2187	−0.9229	0.3724	−0.8463
18:1n-9	−0.6855	−0.0768	−0.7109	−0.1196
16:2n-4	0.6049	0.0746	0.3157	0.2394
18:2n-6	0.1024	−0.0934	0.1855	0.7319
16:3n-4	−0.7364	−0.6384	−0.1794	−0.6668
20:3n-6	−0.9584	−0.1916	−0.3045	0.3926
20:5n-3	−0.9013	−0.4146	−0.8276	−0.4146
N	0.7550	−0.6489		
Total variance, %	37.47	28.85		
Eigenvalue	8.6178	6.6362		

**Table 4:** Fatty acid composition (percentage of total) of *Stephanocyclus meneghinianus* and related species cultured under different nitrogen concentrations.

Species	*Stephanocyclus meneghinianus*				*Chaetoceros muelleri*				*Conticribra weissflogii*		*Conticribra weissflogii*			
Strain	M418		M456						CCMP 1336					
N content, g N/L	0	0.6	0	0.6	0.0025	0.0062	0.0246	0.0494	0.0005	0.0134	0	0.0124	0.0247	0.0494
14:0	7.15	8.09	6.65	6.45	13.89	14.30	14.43	14.60	10.79	10.60	0.13	13.08	8.50	7.46
16:0	47.82	41.91	32.97	29.53	20.48	18.31	17.91	18.50	27.29	22.19	29.11	41.41	30.03	24.26
18:0	1.52	1.04	0.98	0.63	1.80	1.53	1.27	1.49	1.45	1.56	0.81	0.51	0.33	0.29
20:0	0.37	0.41	0.42	0.22		0.12	0.10	0.14			1.39	0.14	0.06	0.14
16:1n-7	35.73	41.72	53.89	58.92	38.39	37.71	37.64	36.62	34.09	25.62	34.98	56.94	42.63	36.39
18:1n-9	0.61	0.42	0.83	0.78	1.42	1.52	1.05	1.40	1.14	2.25	1.08	1.29	0.76	0.58
16:2n-4	0.45	0.52	0.27	0.29					3.22	7.03				
18:2n-6	0.50	0.54	0.81	0.11	1.60	1.33	1.19	1.38		0.16	0.91	0.14	0.38	0.37
16:3n-4	0.80	0.86	0.46	0.53					5.97	9.99				
20:3n-6	0.57	0.41	0.15	0.08	0.24	0.23	0.22	0.21			0.25	0.11	0.10	0.18
20:5n-3	4.48	4.08	2.57	2.46	8.58	9.57	10.55	10.37	10.93	14.71	10.58	14.94	9.98	9.20
SFAs	56.86	51.45	41.02	36.83	41.05	39.50	38.96	39.68	39.53	34.34	41.36	60.36	42.73	35.92
MUFAs	36.34	42.14	54.72	59.70	39.91	39.36	38.81	38.15	35.54	27.87	36.73	58.60	43.73	37.24
PUFAs	6.80	6.41	4.26	3.47	19.03	21.14	22.23	22.17	24.93	37.78	21.91	21.50	14.85	14.39
References	Present study				[35]				[34]		[33]			

The coloring scale illustrates how the relative amounts of main fatty acid groups vary with culturing conditions and differ between species. Coloring scale: from the minimum value (red) to the maximum value (green) within one fatty acid group.

**Table 5:** List of strains with geographic position and description of sample sites.

Strains	Sample Locality	Latitude	Longitude	Substratum	t (°C)	pH	Cond. (mV)
M418	Russia, Moscow, Krylatskoye District, Moskva River	N 55.753740	E 37.448831	Phytoplankton	22.1	7.78	165
M456	Russia, Moscow, Krylatskoye District, Chachenka River	N 55.734546	E 37.335521	Benthos	25.4	7.85	42

## Data Availability

The original contributions presented in this study are included in the article. Further inquiries can be directed to the corresponding authors.

## Supplementary Materials

The following supporting information can be downloaded at: https://www.mdpi.com/article/10.3390/ijms27209030/s1.
