# Supplementary material for: Effect of Nitrogen Concentration on Growth and Fatty Acid Composition in the Freshwater Diatom Stephanocyclus meneghinianus
Source: Int J Mol Sci. 2026 Oct 12;27(20):9030. doi: 10.3390/ijms27209030 (PMC13705416; doi:10.3390/ijms27209030)
Supplement: Supplementary file 1 [file ijms-27-09030-s001.zip › ijms-4592045-supplementary.pdf]

Supplementary Table S1. Fatty acid composition (percentage of total) of *Stephanocyclus meneghinianus* strains M418 and M456 under different cultivation conditions. Values are mean ± SE (n = 3).

**Strain**	**M418**					**M456**				
Experimental variant	WC Nw	WC Nsl	WC Nml	WC Nm	WC Nr	WC Nw	WC Nsl	WC Nml	WC Nm	WC Nr
14:0	7.15±0.06^{Aa}	5.78±0.03^{B}	7.12±0.02^{Ac}	7.31±0.01^{Abd}	8.09±0.01^{C}	6.65±0.06^{D}	6.55±0.08^{D}	6.72±0.09^{D}	6.52±0.04^{D}	6.45±0.04^{D}
16:0	47.82±0.68^{Aa}	44.5±0.61^{Bc}	46.31±0.44^{AB}	50.49±0.51^{ACb}	41.91±0.38^{Bd}	32.97±0.32^{D}	32.26±0.32^{D}	33.64±0.20^{D}	33.32±0.35^{D}	29.53±0.41^{E}
18:0	1.52±0.01^{A}	1.64±0.01^{B}	1.51±0.02^{A}	1.15±0.02^{C}	1.04±0.01^{D}	0.98±0.01^{E}	0.81±0.02^{F}	0.89±0.01^{G}	0.75±0.01^{F}	0.63±0.01^{H}
20:0	0.37±0.01^{A}	0.86±0.01^{B}	0.55±0.02^{C}	0.16±0.01^{D}	0.41±0.01^{E}	0.42±0.01^{E}	0.35±0.01^{A}	0.24±0.02^{F}	0.15±0.01^{D}	0.22±0.02^{F}
16:1n-7	35.73±0.38^{Aa}	38.41±0.46^{B}	37.9±0.40^{Bb}	36.7±0.40^{A}	41.72±0.46^{C}	53.89±0.55^{D}	54.99±0.29^{D}	54.8±0.40^{D}	54.73±0.58^{D}	58.92±0.52^{E}
18:1n-9	0.61±0.01^{A}	0.49±0.01^{B}	0.6±0.01^{A}	0.1±0.01^{C}	0.42±0.01^{D}	0.83±0.01^{E}	0.81±0.02^{Ea}	0.74±0.01^{Fc}	0.75±0.01^{F}	0.78±0.01^{Fbd}
16:2n-4	0.45±0.01^{A}		0.51±0.01^{B}	0.35±0.01^{C}	0.52±0.01^{B}	0.27±0.01^{C}	0.37±0.01^{C}	0.32±0.01^{C}	0.44±0.01^{A}	0.29±0.01^{C}
18:2n-6	0.5±0.01^{A}	0.38±0.01^{B}	0.65±0.01^{C}	0.37±0.01^{D}	0.54±0.01^{A}	0.81±0.02^{E}	0.11±0.01^{F}	0.12±0.01^{F}	0.76±0.02^{E}	0.11±0.01^{F}
16:3n-4	0.8±0.02^{A}	0.95±0.02^{B}	0.77±0.02^{A}	0.56±0.02^{C}	0.86±0.02^{AB}	0.46±0.02^{D}	0.51±0.02^{D}	0.35±0.01^{E}	0.44±0.01^{D}	0.53±0.02^{D}
20:3n-6	0.57±0.02^{A}	0.86±0.02^{B}	0.5±0.01^{A}	0.29±0.01^{C}	0.41±0.02^{D}	0.15±0.01^{E}	0.08±0.01^{F}	0.07±0.01^{F}	0.09±0.01^{F}	0.08±0.01^{F}
20:5n-3	4.48±0.09^{A}	6.13±0.08^{B}	3.58±0.09^{C}	2.52±0.06^{D}	4.08±0.06^{A}	2.57±0.07^{D}	3.16±0.09^{C}	2.11±0.05^{D}	2.05±0.05^{D}	2.46±0.06^{D}
SFAs	56.86±0.73^{A}	52.78±0.62^{B}	55.49±0.46^{A}	59.11±0.53^{A}	51.45±0.37^{B}	41.02±0.28^{C}	39.97±0.26^{C}	41.49±0.31^{C}	40.74±0.39^{C}	36.83±0.44^{D}
MUFAs	36.34±0.38^{Aa}	38.9±0.47^{Eb}	38.5±0.41^{Eb}	36.76±0.40^{Eb}	42.14±0.46^{B}	54.72±0.55^{C}	55.8±0.30^{C}	55.54±0.41^{C}	55.48±0.58^{C}	59.7±0.52^{D}
PUFAs	6.8±0.13^{A}	8.32±0.12^{B}	6.01±0.13^{C}	4.13±0.09^{D}	6.41±0.11^{AC}	4.26±0.09^{D}	4.23±0.10^{D}	2.97±0.07^{E}	3.78±0.08^{F}	3.47±0.08^{F}
omega-3	4.48±0.09^{A}	6.13±0.08^{B}	3.58±0.09^{C}	2.52±0.06^{D}	4.08±0.06^{A}	2.57±0.07^{D}	3.16±0.09^{C}	2.11±0.05^{E}	2.05±0.05^{E}	2.46±0.06^{D}
omega-6	1.07±0.03^{Aa}	1.24±0.03^{B}	1.15±0.03^{AB}	0.7±0.01^{C}	0.95±0.02^{Db}	0.96±0.02^{D}	0.19±0.01^{A}	0.19±0.01^{A}	0.85±0.02^{E}	0.19±0.01^{A}

Different superscript letters show significant differences:

A–H Means within the same line are significantly different with $p \leq 0.01$.

a–d Means within the same line are significantly different with $p \leq 0.05$.
